# Central retina plays a decisive role in the suppression of pupillary escape

**DOI:** 10.1007/s00417-022-05959-1

**Published:** 2023-01-16

**Authors:** Carina Kelbsch, Ricarda Jendritza, Torsten Strasser, Felix Tonagel, Paul Richter, Ronja Jung, Tobias Peters, Helmut Wilhelm, Barbara Wilhelm, Krunoslav Stingl

**Affiliations:** 1 University Eye Hospital, Centre for Ophthalmology, University of Tuebingen, Tübingen, Germany; 2 Pupil Research Group at the Centre for Ophthalmology, University of Tuebingen, Elfriede-Aulhorn-Straße 7, 72076 Tübingen, Germany; 3 Institute for Ophthalmic Research, Centre for Ophthalmology, University of Tuebingen, Tübingen, Germany; 4 Center for Rare Eye Diseases, University of Tuebingen, Tübingen, Germany

**Keywords:** Pupillary escape, Pupil behavior, Pupil light reflex, Central retina, Peripheral retina, Chromatic pupil campimetry

## Abstract

**Purpose:**

To explore the pupil redilation during persistent light exposure (pupillary escape phenomenon) at the macula and periphery with monochromatic light stimuli.

**Methods:**

Forty healthy subjects aged 18–64 years (24 females) were examined by chromatic pupil campimetry (CPC) using red and blue 4-s stimuli of 10° radius at the center and 20°-peripheral locations one per quadrant. One glaucoma patient and one achromatopsia patient served as disease models. For statistical analyses, linear mixed-effects models were performed followed by post hoc *t*-tests.

**Results:**

A distinct pupillary escape could be demonstrated peripherally (blue 0.099%*s, red 0.153%*s); at the central healthy retina, there was no relevant escape, neither for blue nor red stimulation. Comparing central versus peripheral stimulation revealed highly significant differences in the escape (difference blue 0.100 ± 0.013, red 0.144 ± 0.013, < 0.0001, respectively). In the periphery, the escape was significantly more pronounced for red compared with blue stimulation (difference 0.054 ± 0.013; *p* = 0.0001). Enhanced pupillary escape outside of the 95% confidence interval of the linear mixed-effects model of the healthy population could be exemplarily shown in a patient with glaucomatous ganglion cell damage. In the achromatopsia example, no relevant escape was found for blue stimulation, but for red stimulation in the periphery in a comparable range to healthy controls.

**Conclusion:**

The results emphasize that an intact inner retinal network of nerve fibers arising from the central macular region is necessary for maintaining pupillary constriction during a bright 4-s light stimulus and preventing increase of pupillary escape. Increasing receptive field sizes towards the periphery on the level of retinal ganglion cells and less input from central 1:1 connections could be one of the driving mechanisms for pupillary escape.

**Supplementary Information:**

The online version contains supplementary material available at 10.1007/s00417-022-05959-1.


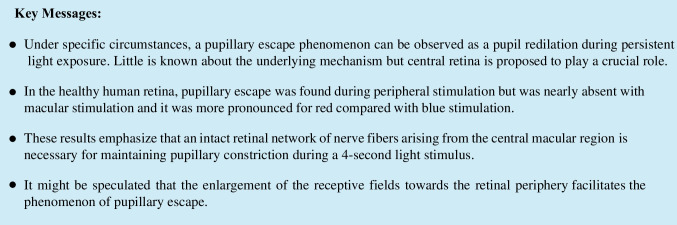


## Introduction

A pupillary escape phenomenon (in the following “pupillary escape”) means that the pupil begins to redilate during constant light exposure after the initial phasic contraction to a light stimulus.

This term is sometimes also used for a phenomenon observed with the swinging-flashlight test: In case of a marked relative afferent pupillary defect, the pupil will dilate when the flashlight swings from the better eye to the eye with the afferent defect. This is not meant here.

Our study is about the “escape” of the pupil that has been “trapped” by a constant light stimulus. One might expect that the pupil remains constricted as long as the stimulus is present and dilates when the stimulus is switched off. However, under specific circumstances, pupillary dilation begins earlier. Little is known about the underlying mechanism of this pupillary behavior although being described since decades. In Loewenfeld’s comprehensive textbook about the pupil’s anatomy, physiology, and clinical applications based on cooperative work with Otto Lowenstein [1], the pupillary escape is described as a normal pupillary behavior in response to light stimuli of low intensity and a pathological behavior to light stimuli of high intensity in patients with lesions in the retina or afferent pathways.

In a previous study of our group, using full-field color pupillography in samples of healthy eyes, glaucomatous eyes, and eyes with ocular hypertension [2], pupillary escape could be observed in many glaucoma patients and two single patients with ocular hypertension during stimulation with a 28-lx red 4-s stimulus. The slope of redilation as the parameter of pupillary escape was statistically significantly steeper for glaucoma patients compared with the control group (glaucoma 2.40 ± 1.81 versus control group 0.04 ± 1.54, *p* < 0.0005) and correlated positively with the extent of the visual field defects. A higher susceptibility for a conversion from ocular hypertension into manifest glaucoma for individuals with a more pronounced escape was assumed, and altered interactions between different photoreceptors and the afferent neuroretinal network were proposed. Bergamin and Kardon [3] analyzed pupillary escape as the ratio of the sustained to the initial phasic pupil response in patients with central, peripheral, or combined visual field loss using full-field light stimuli of 5-s duration. They found a statistical difference between those with central visual field loss and normal eyes but not between those with only peripheral visual field loss and normal eyes and concluded that afferent neurons from the central retina are essential for normal sustained pupil responses.

The aim of this study was to gain more insight into this mysterious pupillary escape and its clinical applicability by analyzing pupillary responses to red and blue stimulation in a group of healthy participants. In contrast to the full-field stimulation paradigm used in the aforementioned studies, chromatic pupil campimetry (CPC) was used that enables a local retinal stimulation. Pupillary responses to central macular stimulation were compared with those obtained from stimulation more peripherally in the four retinal quadrants for red and blue stimulation, respectively.

## Methods

### Participants

Forty adult volunteers aged 18–64 years (mean 39 years, 16 males, 24 females) participated in the study. The cohort represented a subgroup of a project for establishing a normative database for cone-specific and rod-specific protocols of CPC, which has been approved by the local institutional ethics committee and followed the tenets of the Declaration of Helsinki.

Additionally, one glaucoma patient and one achromatopsia patient were examined as exemplary disease models.

All participants underwent an detailed ophthalmological examination prior to study participation, including history, best-corrected visual acuity (BCVA), pupil testing, slit lamp examination of anterior segments and fundus ophthalmoscopy, visual field testing (30° static perimetry with Octopus 900, Haag-Streit International, Wedel, Germany), and optical coherence tomography (Spectralis-OCT, Heidelberg Engineering GmbH, Heidelberg, Germany, standard ring scan of the optic nerve and macula volume).

Exclusion criteria were any history or clinical findings of eye diseases, systemic disorders, and/or medication influencing pupil reactivity or retinal function or interfering with the measurement (e.g., nystagmus). BCVA was at least 1.0 decimal in all participants. Refraction error was limited to a spherical equivalent of +/− 3 D.

### CPC

Pupillary responses were elicited and analyzed by using CPC (custom-built pupillograph; stimulus presentation on an OLED monitor (LG OLED 55C7V; LG Electronics, Seoul, South Korea; Full HD 3840 × 2160 pixels), precise continuous pupil recording via an infrared camera (DMK23UV024, The Imaging Source GmbH; with a 50-mm TV-lens 1:1.8; temporal resolution 10 ms)). Pupil size was calculated in real time by an in-house software as described before in detail [4]. The examination was performed in a quiet room in complete darkness. As a fixation target, a dim central fixation mark at the monitor (0.01 cd/m^2^) was used. Gaze-tracking correcting for subtle eye movements at stimulus initialization ensured further a retinotopic stimulus presentation. While one eye was patched, the direct pupillary response of the other eye was recorded. The method has been validated in a healthy cohort including good test–retest reliability profile [5] and several retinal diseases [6–8] before.

**Fig. 1:**
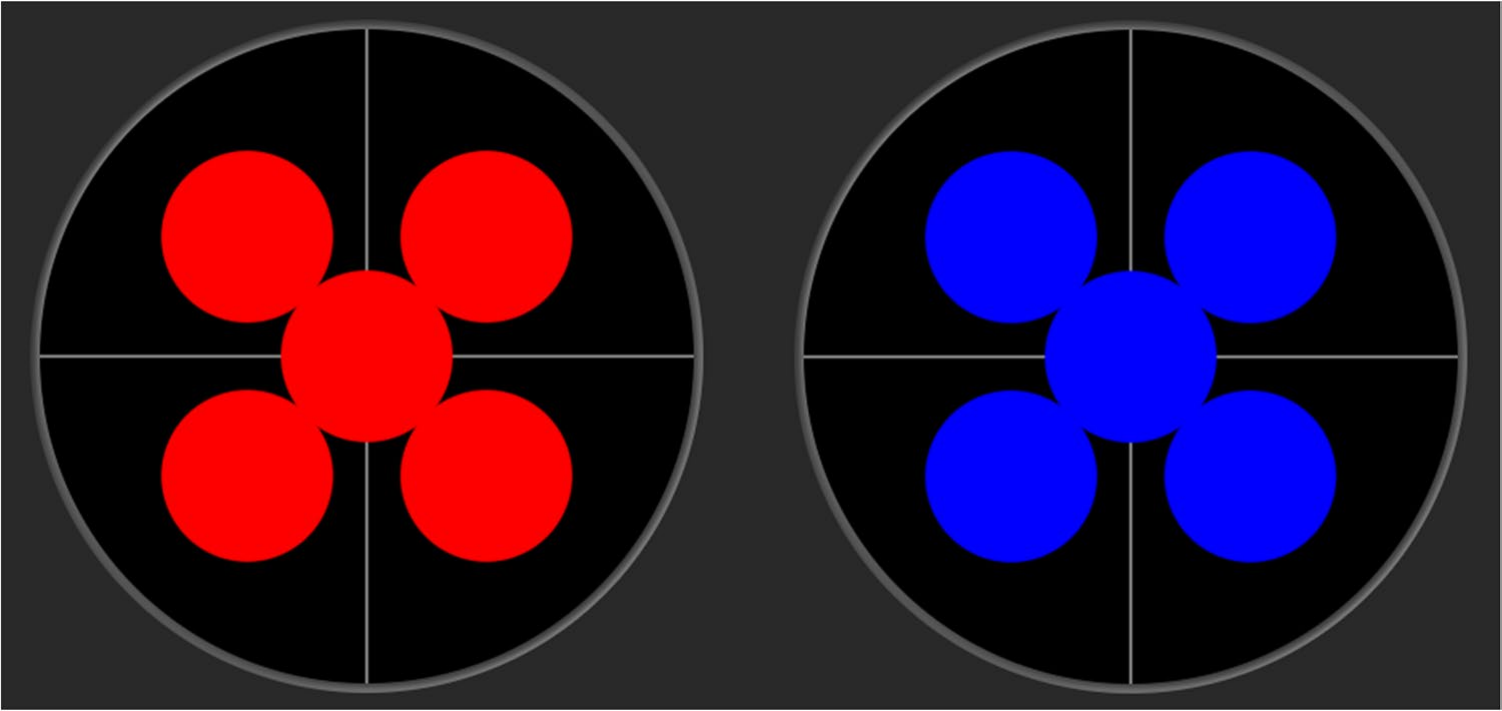
Stimulus grid. Stimuli were presented one after the other at 5 different locations: centrally at 0° and peripherally at 20° eccentricity in each quadrant; stimuli radii 10°; at least three repetitions per location; first run with red stimuli and second run with blue stimuli

Stimuli were presented on the OLED monitor at a distance of 40 cm to the subject’s eye at the fovea and at 20° eccentricity of the four retinal quadrants, respectively. The stimulus grid can be seen in Fig. 1. Stimulus characteristics were chosen in accordance with current standards in pupillography [9]. Stimulus size was 10° radius, stimulus duration 4 s, baseline period before stimulus presentation 500 ms, and interstimulus interval 7.5 s (from the end of one stimulus until the beginning of the next stimulus). Stimulus wavelength was either 620 ± 30 nm FWHM (red) or 460 nm ± 30 nm FWHM (blue) with a stimulus intensity of 60 cd/m^2^ for red stimulation (irradiance 3.3 × 10^−2^ W/m^2^; peak intensity 0.590 mW/m^2^) or 20 cd/m^2^ for blue stimulation (irradiance 4.6 × 10^−2^ W/m^2^, peak intensity 1.319 mW/m^2^). Stimuli were presented in complete darkness without background lightening, subsequently after having performed the cone-specific protocol (red stimuli of 60 cd/m^2^ on a dim blue background of 0.01 cd/m^2^) for the normative database establishment. There was no prior dark-adaptation. More details about the stimulation properties with regard to energy and spectrum including CIE color coordinates of the applied stimuli are shown in Online Supplementary Figure 1. Out of three repetitions per stimulus location, the average pupillary response was calculated. A stimulus was automatically repeated by the software in case of blinking artifacts during stimulus presentation, or if at least 90% of the baseline diameter was not reached before the next stimulus presentation.

### Data analysis and statistics

Artifact elimination was performed by linear interpolation with an in-house created software (The MathWorks, Inc Matlab, for more details: Stingl et al 2018 [4]) and manually checked. All measured left eyes were mirrow-converted to right eyes.

For the statistical analysis, three pupillary response parameters were determined: the relative maximal constriction amplitude (relMCA; normalized to baseline diameter in percent), the latency to constriction onset (latency; in ms; calculated as the time from the beginning of stimulus presentation until the time of intersection of linear fitting curves through the descending part of the pupillogram and the baseline, respectively), and the escape, a pupillary redilation during stimulus light exposure. The escape was calculated as the integrated area under the curve of the pupillogram referenced to the horizontal line at maximal constriction in the time span 2–4.5 s (end of stimulus) according to the formula $\sum_{2\ s}^{4,5\ s}\left(\textrm{rel}.\textrm{pupil}\ \textrm{diameter}-\textrm{minimal}\ \textrm{rel}.\textrm{pupil}\ \textrm{diameter}\ \textrm{of}\ \textrm{the}\ \textrm{interval}\ 1\ \textrm{to}\ 2\ \textrm{s}\right)\ast 0.01$ (%*s). Online Supplementary Figure 2 visualizes the calculation of pupillary escape.

For relMCA, latency, and escape, a linear mixed-effects model was performed for statistical analyses with the fixed-effects stimulus color (red versus blue) and stimulus location (center versus periphery) and the subject as random effect, followed by post hoc *t*-tests. Assumptions for linear mixed models were checked and assumed. As one limitation, homogeneity of the residual variances for the escape parameter could not be verified in the data set. However, it could be proven that mixed-effects models show remarkable robustness and can be used even if the distributional assumptions are violated as the dataset keeps unbiased [10].

Results are presented in least square (LS) mean values, difference ± standard error (SE), and confidence intervals (CI). To correct for multiple comparisons in the post hoc analyses, the alpha level was set to 0.0125 (Bonferroni correction).

## Results

**Fig. 2:**
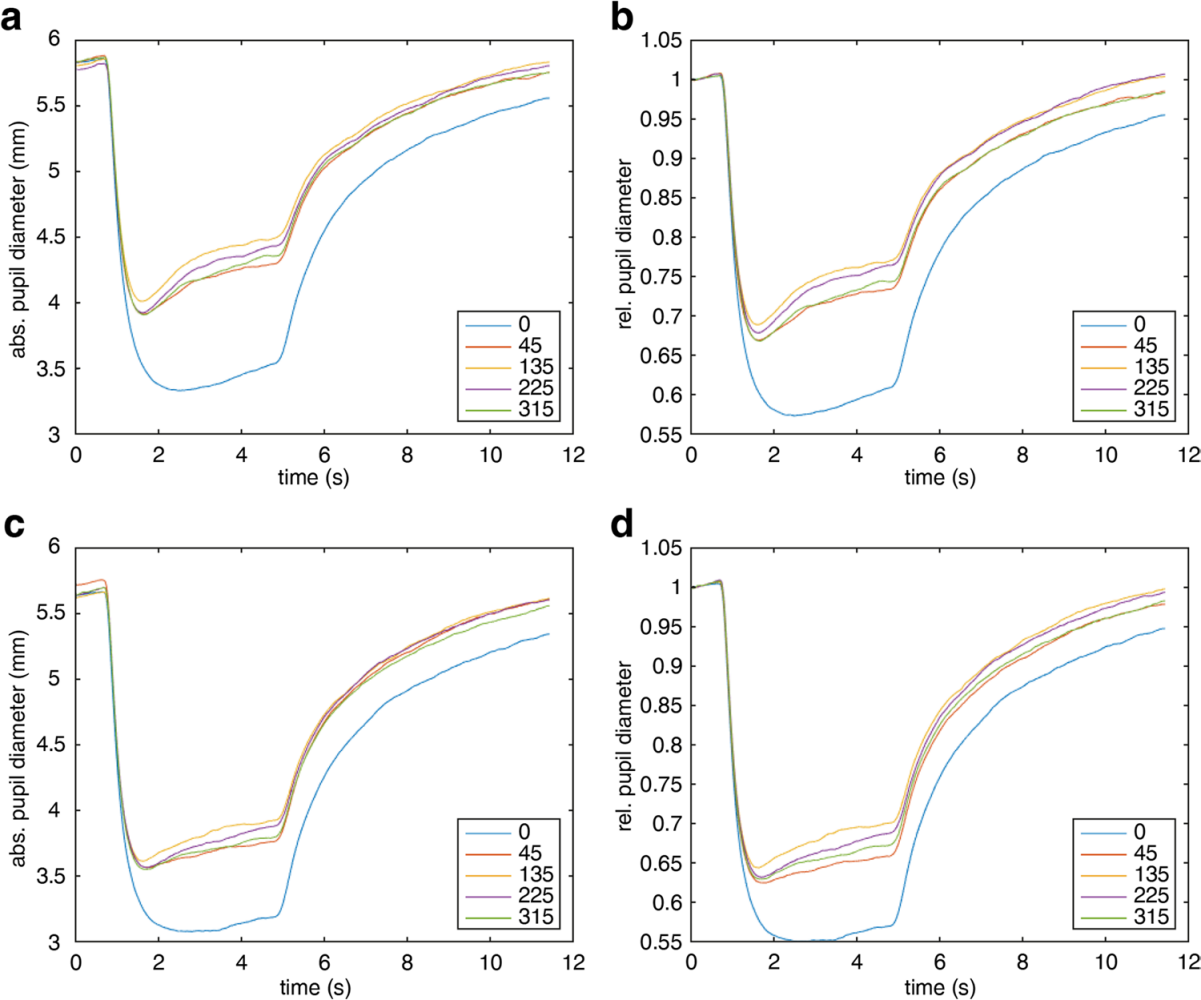
Averaged pupil traces of all participants (*n* = 40) in absolute values (**a, c**) and relative values normalized to baseline (**b, d**) for red stimulation (**a, b**) and blue stimulation (**c, d**). Traces are shown for stimulation at the center 0° and 20° periphery of all four quadrants (45 = temporal upper visual field; 135 = nasal upper visual field; 225 = nasal lower visual field; 315 = temporal lower visual field). Stimulus duration was 4 s from 0.5 to 4.5 s

Figure 2 shows the mean pupillary traces of all 40 participants in absolute values and relative values normalized to baseline for blue and red stimulation at the center and the periphery of all four retinal quadrants.

### relMCA

**Fig. 3:**
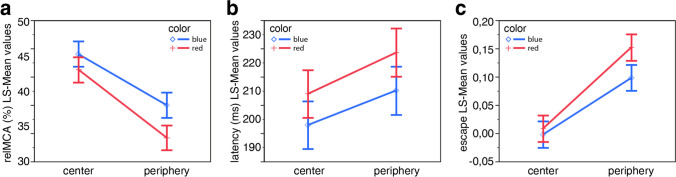
Pupillary response parameters in least square (LS) mean values and confidence intervals by linear mixed models for central versus peripheral stimulation with blue and red stimuli, respectively: relMCA (in %) (**a**), latency to constriction onset (in ms) (**b**), and escape as a parameter for pupil redilation during ongoing light stimulation (%*s, integrated area under the pupillogramm referenced to the horizontal line at maximal constriction in the time span 2–4.5 s (end of stimulus); for more details, see the “Methods” section and Supplementary Fig.2) (**c**).

relMCA was significantly more pronounced in the center than in the periphery for both red stimulation (center 43.13%; periphery 33.44%; difference 9.69 ± 0.65; CI 8.41, 10.97; *p* < 0.0001) and blue stimulation (center 45.32%; periphery 38.07%; difference 7.25 ± 0.65; CI 5.97, 8.54; *p* < 0.0001). Moreover, there was a statistically significant difference of relMCAs between blue and red stimulations (center difference 2.20 ± 0.65; CI 0.92, 3.48, *p* = 0.0009; periphery difference 4.63 ± 0.65; CI 3.35, 5.91, *p* < 0.0001) (see Fig. 3a).

### Latency to constriction onset

Shorter latencies were found for central versus peripheral stimulation for both colors and for blue versus red stimulation (center blue 198 ms, periphery blue 210 ms; center red 209 ms, periphery red 224 ms; SE of differences 2.63; all *p* < 0.0001) (see Fig. 3b).

### Pupillary escape

Pupillary escape could be demonstrated in the periphery; at the central retina, there was no relevant escape, neither for blue nor for red stimulation (center blue − 0.002%*s, center red 0.009%*s; difference 0.011 ± 0.013; CI -0.016, 0.037; no significant difference, *p* = 0.43). Comparing central versus peripheral stimulation consequently revealed highly significant differences in pupillary escape for both colors (difference red 0.144 ± 0.013, difference blue 0.100 ± 0.013, *p* < 0.0001, respectively). In the retinal periphery, pupillary escape was significantly more pronounced for red stimulation (0.153%*s) compared with blue stimulation (0.099%*s) (difference 0.054 ± 0.013; CI 0.027, 0.081; *p* = 0.0001) (see Fig. 3c and Table 1). Between the quadrants of one color, no significant difference was found.

**Table 1:** Enhanced pupillary escape (%*s) for both eyes (R/L) of a patient with progressive glaucomatous damage in comparison to the least square (LS) mean values and 95% confidence intervals (CI) of the linear mixed model for healthy participants (*n* = 40). Standard error = 0.012. The achromatopsia patient (ACHM) revealed no relevant escape for blue stimulation and relatively comparable results to healthy participants of a slight pupillary escape for red stimulation in the periphery

	Center	Periphery
Red		
Glaucoma	R 0.231/L 0.303	R 0.398/L 0.430
Healthy	0.009, CI − 0.014, 0.032	0.153, CI 0.130, 0.176
ACHM	R 0.034/L 0.114	R 0.120/L 0.153
Blue		
Glaucoma	R 0.252/L 0.164	R 0.287/L 0.290
Healthy	− 0.002, CI − 0.025, 0.021	0.099, CI 0.076, 0.122
ACHM	R − 0.012/L 0.074	R 0.037/L 0.051

### Exemplary results of a patient with achromatopsia and a patient with glaucoma

As a proof of concept of hypotheses and interpretations of the results, an achromatopsia patient (ACHM) with *CNGA3* mutations (c.1585G>A ➔ p.Val529Met and c.1641C>A ➔ p.Phe547Leu heterozygous, respectively) served as an example of non-functioning cones, and a patient with progressive glaucoma as an example of ganglion cell damage with preserved photoreceptors.

**Fig. 4:**
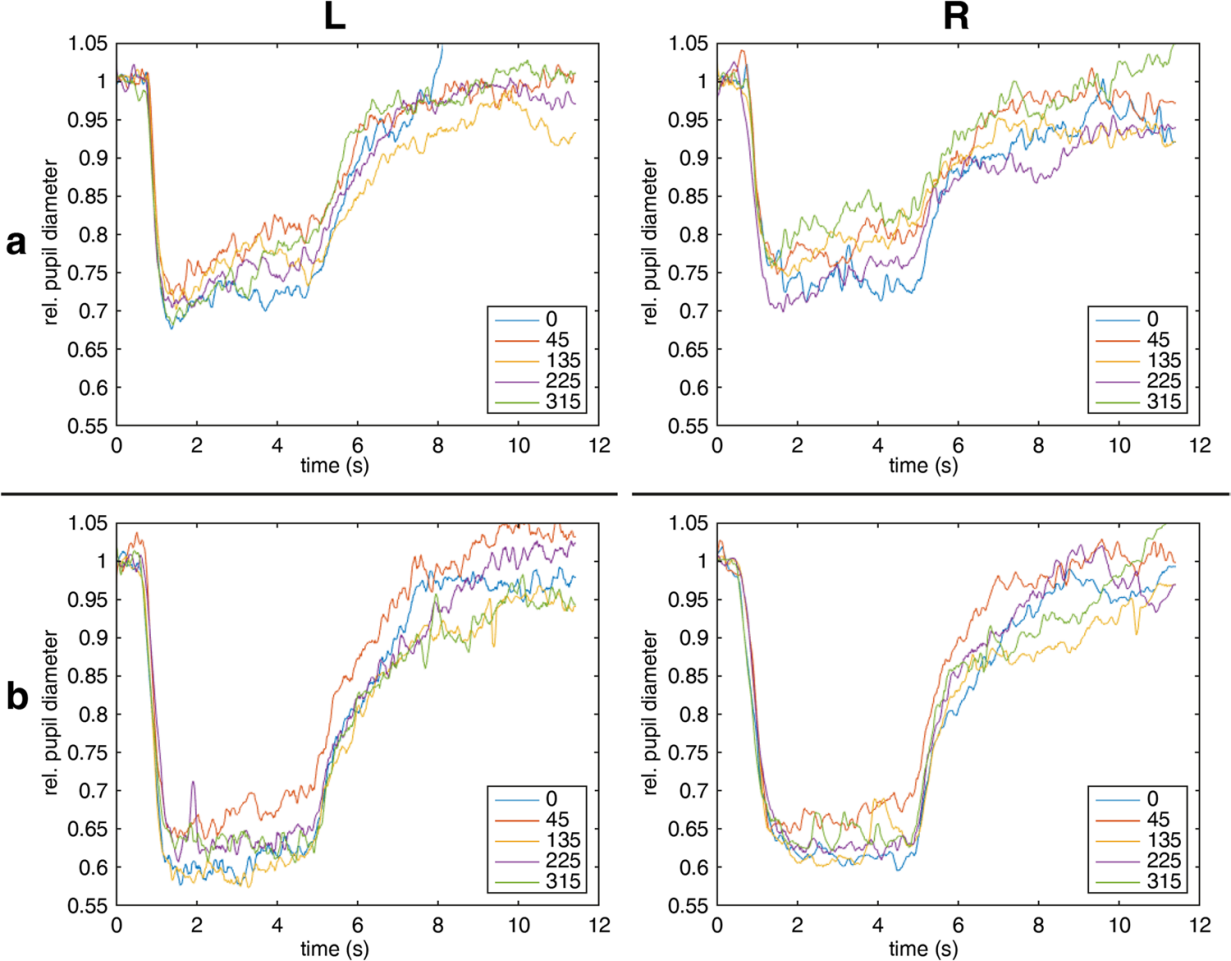
Pupil traces normalized to baseline from an achromatopsia patient with non-functioning cones due to *CNGA3*-mutations reveal no pathological escape phenomenon (**a** red stimulation; **b** blue stimulation). Results from left eye (L) are shown in the left and from right eye (R) in the right column

The ACHM patient had a best-corrected visual acuity of 0.05 decimal in both eyes and a typical slight nystagmus. Fundus ophthalmoscopy was unremarkable; the electroretinogram revealed preserved rod responses and no cone responses. In ACHM, no relevant pupillary escape could be observed in CPC for blue stimulation, but for red stimulation, pupillary escape was found to be in a comparable range to the healthy control group in the periphery (see Fig. 4 and Table 1).

**Fig. 5:**
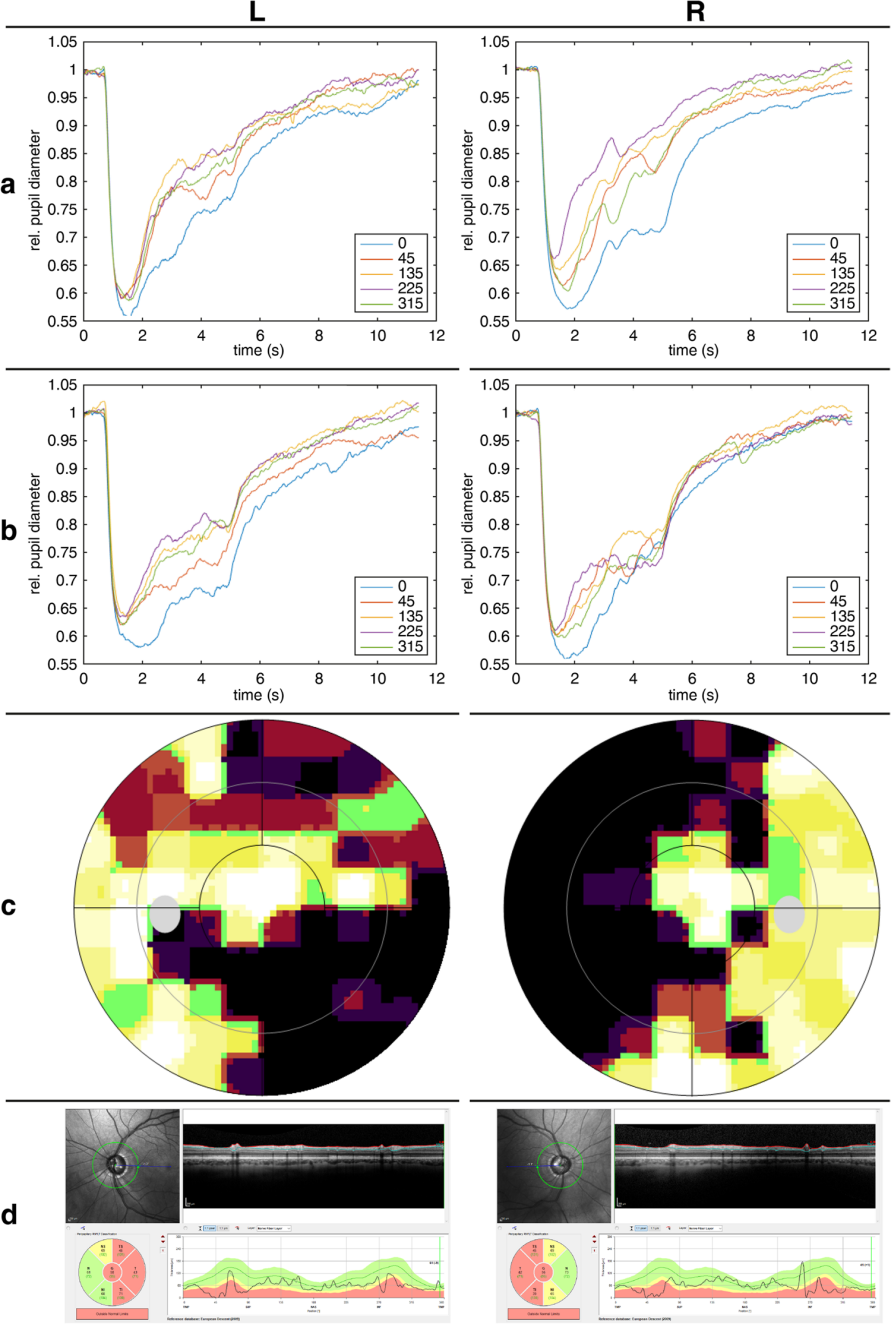
Example of a glaucoma patient with ganglion cell damage of the optic nerve. Averaged pupil traces normalized to baseline from both eyes reveal a clear pupillary escape, a redilation during light stimulation to a 4-s stimulus (**a** red stimulation; **b** blue stimulation) not only to peripheral stimulation as in healthy participants, but also in the center, particularly during red but also during blue stimulation. Clinical findings of visual field defects (30° Octopus 900, Haag-Streit) (**c**) and reduced peripapillary nerve fiber layer thickness in Spectralis-OCT (**d**) are shown, respectively. Results from left eye (L) are shown in the left and from right eye (R) in the right column

The glaucoma patient suffered from normal-pressure glaucoma with a glaucomatous cup-disc ratio of 0.9 on both eyes, visual field defects, and reduced peripapillary nerve fiber layer thickness in OCT with a preserved visual acuity of 1.0 decimal in both eyes. Clinical findings and pupillary responses from CPC are shown in Fig. 5. In contrast to healthy subjects, an enhanced pupillary escape phenomenon was present not only during peripheral stimulation but also during central stimulation in the glaucoma patient for both wavelengths. The results of the escape of the glaucoma patient laid outside of the confidence interval of the least square mean values of the linear mixed model for the healthy population, thus with 95% indeed outside the normal mean, as can be seen in Table 1.

## Discussion

The present study is the first to compare the influence of central versus peripheral red and blue stimulation on the pupillary escape phenomenon in the healthy retina.

Our results indicate that a retinal network of nerve fibers arising from the central macular region is necessary for maintaining pupillary constriction during a bright 4-s light stimulus and preventing increase of pupillary escape. This hypothesis is supported by the results of a distinct pupillary escape being found during peripheral stimulation but only in a negligible amount in the macula in the healthy human retina. Moreover, an enhanced peripheral and distinct central pupillary escape could be exemplarily shown in a patient with glaucomatous ganglion cell loss in both eyes. However, in achromatopsia, no abnormal pupillary escape was found.

We observed a significantly more pronounced pupillary escape for red stimulation in the periphery of the healthy retina than for blue stimulation (red 0.153%*s compared with blue 0.099%*s, *p* = 0.0001). This observation was already noticeable in our group’s previous study, where pupillary escape was exclusively shown for red, but not for blue 28-lx full-field stimulation [2]. With the current local stimulation paradigm in CPC, the pupillary escape was barely present centrally, neither for red nor for blue stimulation, but in the periphery particularly for red stimulation.

Different pathways for red and blue stimulation may explain these findings. In the healthy retina, red stimulation is targeting predominantly (M-/) L-cones that synapse with ON/OFF midget bipolar cells and subsequent midget retinal ganglion cells (RGCs) and reach the parvocellular layers of the dorsal lateral geniculate nucleus (dLGN) before connecting to layer 4 of the primary visual cortex (V1). Blue stimulation, however, is predominantly activating S-cones and melanopsin of the intrinsically photosensitive retinal ganglion cells (ipRGCs). S-cones connect via S-ON bipolar cells and small bistratified RGCs to the koniocellular layers of the dLGN and layers 1 and 3 of V1 and via S-OFF midget bipolar cells to OFF midget RGCs [11–14]. Furthermore, all cones connect synaptically with ipRGCs that play a decisive role in the pupillary light reaction by projecting to the olivary pretectal nucleus in the dorsal midbrain [15–17], the controlling center of the pupillary light reaction. Different synaptic connections for L-cones and S-cones to ipRGCs are evident. Recently, it could be shown in the primate retina that S-cones contact via S-ON bipolar cells not only to the small bistratified RGCs but also to S-cone-specific amacrine cells that in turn synapse in an inhibitory manner with M1 ipRGCs [18]. Thus, there is an exclusive S-cone- pathway to ipRGCs, at least in the primate retina. Furthermore, ipRGCs might to some extent be directly activated by our blue stimulus, although the stimulus characteristics were not designed as melanopsin-specific which probably would have required higher intensities [9, 19], but wavelength, stimulus duration, and dark background were potentially adequate for melanopsin excitation. However, in our data, no extensive post-illumination pupil response is obvious, presumably indicating no substantial melanopsin activation.

Thus, different cell pathways for red and blue stimulation might explain the different extent of evoked pupillary escape.

Cones in general show a peak density at the fovea of around 150,000–200,000 cells/mm^2^, drop rapidly to around 15,000 cells/mm^2^ at 1 mm eccentricity, and gradually further decline to around 3500 cells/mm^2^ outside 12 mm eccentricity. S-cones, that represent around 6–10% of all cones, however, are nearly absent in the central fovea and show a density peak of 2400 cells/mm^2^ at 0.36 mm eccentricity [20].

Cone cell density and their 1:1 interconnection in the macula with enlarging receptive fields towards periphery might be another relevant factor in understanding the pupillary escape phenomenon.

It could be previously shown by immunohistochemical studies in human retinae that the ratio of cones to OFF-midget bipolar cells of the parvocellular pathway is similar and indicates a 1:1 connectivity at least to ~ 35° retinal eccentricity. Midget ganglion cell density, however, showed a much steeper decline from the center to the periphery, thus resulting in convergence of multiple midget bipolar cells to one midget ganglion cell [20]. The averaged density of midget ganglion cells decreased below that of cones around 9° eccentricity. Consequently, it might be speculated that the enlargement of the receptive fields towards the periphery on the level of ganglion cells and less input from central 1:1 connections facilitates the occurrence of a pupillary escape phenomenon. Likewise, degeneration on the level of RGCs, as it is the case in glaucoma, would result in enhanced pupillary escape behavior, as could be exemplarily shown in both eyes of our glaucoma patient. This is also in line with the results of our previous study with full-field stimulation (and thus including the macula), revealing a significant pupillary escape in the cohort of glaucoma patients compared with healthy participants [2] and the escape being positively correlated with the extent of the visual field defects. Likewise, the results are consistent with the aforementioned results from Bergamin and Kardon [3] showing a significant pupillary escape only in central visual field defects compared with normals, but not in peripheral defects. Reversely, although not being designed to examine pupillary escape, experiment 1 by Park et al. [19] revealed no relevant pupillary escape for full-field stimulation in healthy participants, as would be expected from our hypothesis that the central retina plays a decisive role in the suppression of pupillary escape.

In accordance with former pupillographic studies with full-field stimulation in a cohort of *CNGA3*-linked ACHM [21], the illustratively presented ACHM patient still revealed relatively good pupillary responses although having heavily impaired cone function. Several factors are considered to contribute, including a hypersensitivity of rods and ipRGCs in the absence of functioning inhibiting cones. It was shown that cone bipolar cells arrange ectopic synapses with neighboring rods in the absence of normal cone input [22]. Consequently, the rearrangement of the neuronal retinal network with typically preserved inner retina in ACHM might explain that no pathological pupillary escape could be found in the ACHM patient. In advanced retinitis pigmentosa (RP), however, an inherited rod-cone dystrophy, secondary damage to the inner retina after photoreceptor decline, usually occurs over time. Pupillographic data from a previous study of our group in a big cohort of late-stage RP reveal retrospectively pupillary escape to red full-field stimulation [23], again indicating a decisive role of an intact retinal network from the outer to the inner central retina to prevent the occurrence of a pupillary escape.

Furthermore, as expected, relMCA was stronger for central versus peripheral stimulation. Latencies were shorter centrally compared with the periphery with the long-lasting, high-intensity stimulation used in this study. In a different stimulation paradigm with increasing stimulus sizes of circle and ring stimuli of lower duration and intensity, an important role of the central retina on relMCA was accordingly shown in a previous study of our group. However, a particular relevance of peripheral retinal stimulation on pupillary latencies was hypothesized [24]. This probably was an effect of stimulus shape and size, as shorter latencies were obtained in the present study for central versus peripheral stimulation when stimulus size and intensity were equally matched.

## Limitations

Each calculation of pupillary escape has its limits, and this is also true for our selection of period and method of escape quantification. We consider the chosen paradigm as the least error-prone; however, we are completely aware that it is likewise a compromise. Slight underestimation of the amount of pupillary escape occurs in cases of (a) very fast or very slow pupillary reactions when maximal constriction is not falling into the expected interval from 1 to 2 s and (b) fast responders in whom relMCA is reached very early in the expected interval of 1–2 s as the integrated area under the curve is purposely kept constant from 2–4,5 s. However, these minor effects should not affect the results and the interpretation in a relevant way as it applies systemically for both central and peripheral stimulation.

This study was designed as an explorative study in healthy human retinae to gain more insight into physiological pupillary responses and retinal interconnections. Such knowledge becomes more and more important as pupillary responses have the great potential serving as objective biomarkers for retinal function in current and up-coming interventions like gene therapy or retinal implants. However, statements about the mechanism of pupillary escape and the interconnection of the retinal network remain of hypothetical nature. Furthermore, inclusion of one glaucoma and one achromatopsia patient was used for illustrative purposes and as indication that this type of measurement could have an interesting future in clinical setup. However, no bigger patient cohorts were included to provide statistical power. We hope that this study encourages for future studies targeting these questions.

## Supplementary information

ESM 1 (PDF 215 kb)
