# Supplementary material for: Central retina plays a decisive role in the suppression of pupillary escape
Source: Graefes Arch Clin Exp Ophthalmol. 2023 Jan 16;261(6):1713–22. doi: 10.1007/s00417-022-05959-1 (PMC10199107; doi:10.1007/s00417-022-05959-1)
Supplement: Supplementary file 1 — (PDF 215 kb) [file 417_2022_5959_MOESM1_ESM.pdf]

Supplementary Material

to

Graefe's Archive for Clinical and Experimental Ophthalmology

Title: Central retina plays a decisive role in the suppression of pupillary escape

Authors: Carina Kelbsch[1,2] , Ricarda Jendritza[2], Torsten Strasser[1,2,4], Felix Tonagel[1], Paul Richter[1,2], Ronja Jung[1,2], Tobias Peters[2], Helmut Wilhelm[1,2], Barbara Wilhelm[2,*], Krunoslav Stingl[1,2,3]

Affiliations:
[1] University Eye Hospital, Centre for Ophthalmology, University of Tuebingen, Germany
[2] Pupil Research Group at the Centre for Ophthalmology, University of Tuebingen, Germany
[3] Center for Rare Eye Diseases, University of Tuebingen, Germany
[4] Institute for Ophthalmic Research, Centre for Ophthalmology, University of Tuebingen, Germany

*Corresponding author
Email: Barbara.wilhelm@stz-eyetrial.de

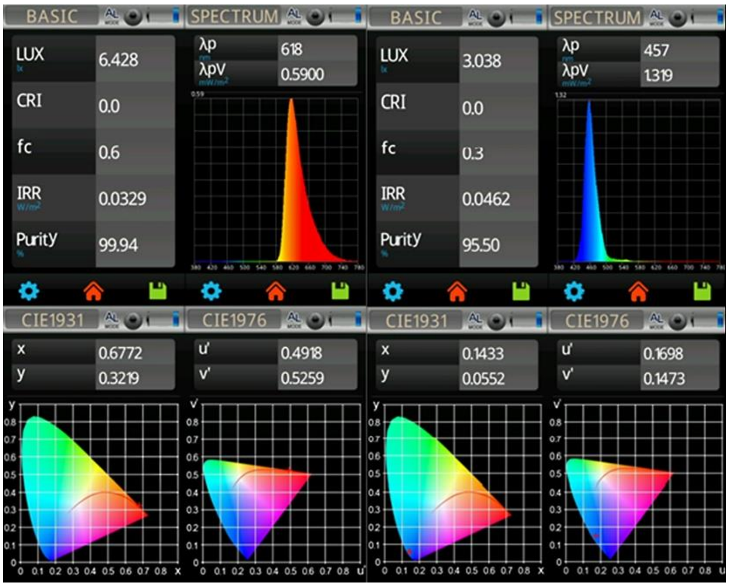

Supplementary Figure 1 Energy and spectrum with CIE color coordinates of the applied stimuli.

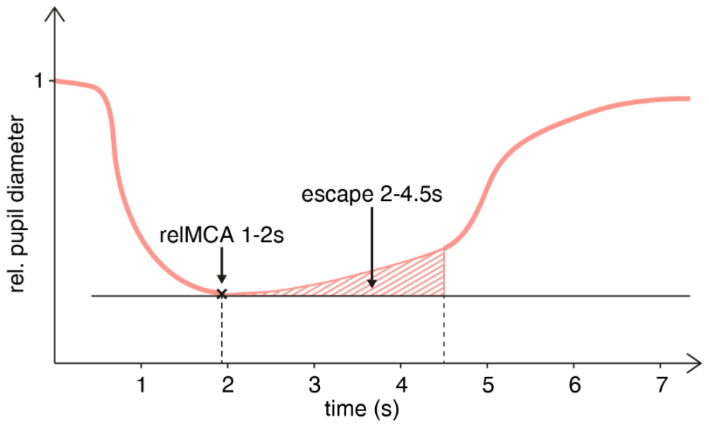

Supplementary Figure 2 Calculation of pupillary escape as the integrated area under the curve of the pupillogram referenced to the horizontal line at maximal constriction in the time span 2 – 4.5 s.
